# Modes of action and emerging strategies in next-generation fungicide development against phytopathogenic fungi

**DOI:** 10.3389/fpls.2026.1906086

**Published:** 2026-10-05

**Authors:** Jongchan Woo, Seungmee Jung, Eunsook Park

**Affiliations:** 1 Department of Molecular Biology, College of Agriculture, Life Sciences and Natural Resources, University of Wyoming, Laramie, WY, United States; 2 Innocorelix, LLC., Laramie, WY, United States

**Keywords:** autophagy, fungicide resistance, fungicides, new target development, plant fungi

## Abstract

Plant fungal diseases impose significant annual reductions of global crop yield and quality. Despite advances in our understanding of the etiology and molecular mechanisms of plant-fungus interactions, effective management of fungal diseases remains challenging due to climate-driven shifts in pathogen populations, the emergence of fungicide-resistant strains, globalization in agricultural supply chains, and postharvest vulnerabilities. To address these challenges, research in plant pathology has leveraged state-of-the-art technologies, with the development of novel fungicides serving as a major step forward in crop protection. This review highlights advances in fungicide development against pathogenic fungi, with an emphasis on the modes of action and regulatory mechanisms of commercial active ingredients that reflect current trends in efforts to secure global food resources. Furthermore, emerging technologies and innovative approaches for developing next-generation fungicides are discussed. By leveraging mechanistic insights and applied innovations, future research can be guided to enable the rational design of fungicides to mitigate both current and emerging agricultural challenges.

## 1 Introduction

Although global food production has been revolutionized over the past half century, it is increasingly challenged by a combination of biotic and abiotic factors whose interactions create vulnerable conditions in arable systems. Rising temperature and environmental variability not only reshape the population dynamics and adaptive capacity of pathogenic species but also facilitate their range expansion into novel ecological niches. This shift accelerates the emergence and dissemination of novel pathogens, posing a significant threat to global food security. Fungal pathogens cause substantial agricultural product spoilage through direct infection and the subsequent accumulation of mycotoxins. While the globalization of the food supply chain of agricultural products supports global nutritional sustainability, it simultaneously serves as a vector for the introduction of exotic pathogens into previously naïve geographic regions through the consumption of contaminated commodities (Ristaino et al., 2021). These compounded stresses impose substantial constraints on crop productivity and quality. Devastating plant pathogens destroy major crops estimated to be approximately 22% in maize, 17% in potato, 30% in rice, 21% in soybean, and 21% in wheat, corresponding to a global economic impact of nearly $220 billion annually (Savary et al., 2019; Wang et al., 2024). Current plant disease management relies on cultural practices, host resistance, biological control, and chemical fungicides, often integrated within disease management frameworks. Despite advances in understanding pathogen etiology and in scientific discoveries and technologies that help mitigate crop losses, plant disease management strategies have remained largely unchanged over the past decades (Beckerman et al., 2023). This innovation gap is attributed to the limited development and deployment of novel biological and chemical control strategies. Therefore, there is an urgent need to develop new management solutions including next-generation fungicides.

Emerging evidence supports the importance of a multidisciplinary approach integrating scientific knowledge, state-of-the-art technologies, and expertise from diverse research and field disciplines for the effective management of plant pathogen outbreaks (Jeger et al., 2021). Despite their profound impact on global food security, exemplified by the catastrophic Panama disease that wiped out the ‘Gros Michel’ banana, plant disease outbreaks remain underprioritized compared to pandemic outbreak of human disease (Ristaino et al., 2021). Although plant-microbe interaction studies have elucidated key mechanisms of pathogenesis, their translation into field-deployable detection and forecasting systems is still limited. Integrating pathogen surveillance data with atmospheric parameters and analyzing these datasets using artificial intelligence (AI) enable predictive, data-driven models of disease outbreaks across landscapes. However, current disease monitoring remains fragmented, limited to a narrow range of pathogens, and largely restricted to developed countries. This situation underscores the urgent need for real-time disease surveillance, standardized data sharing, and international collaboration to integrate information on plant disease outbreaks, enabling reliable forecasting that can be effectively incorporated into disease management programs (Carvajal-Yepes et al., 2019). Such coordinated and systemic responses have the potential to substantially improve both the efficiency and resilience of agricultural production.

Fungicides serve as a central tool for disease control. Fungicide resistance as emphasized by Delp and Dekker (Delp and Dekker, 1985), refers to the stable and heritable adaptation of fungal pathogens to fungicidal compounds. This phenomenon typically arises from mutations in the fungicide target site, resulting in significantly decreased susceptibility. Early fungicides, particularly sulfur- and copper-based formulations in the early 20^th^ century functioned as multisite inhibitors and were frequently applied at high rates (Morton and Staub, 2008). Although these compounds exhibit lower efficacy than later-developed systemic fungicides, the emergence of resistant fungal pathogens to these formulations has remained sporadic (Yin et al., 2023). In general, this observation suggests that multisite modes of action impose stronger constraints on pathogen adaptation and resistance development than the selective and site-specific modes of action of modern fungicides (Leadbeater, 2015). In contrast, target-specific fungicides act on a single pathway essential for fundamental fungal development and pathogenicity, enabling effective control at lower application rates. Because they reduce off-target effects on the microbiome and the surrounding environment compared with the multisite fungicides, most modern fungicide development has shifted toward target-specific modes of action. Despite the widespread use of commercially available fungicides, not all modes of action have been characterized, and their associated off-target and ecological impacts are still insufficiently understood (Yang et al., 2011; Zubrod et al., 2019; Noel et al., 2022), suggesting limitations in reliably predicting the broader environmental and agronomic consequences of fungicide application. Nevertheless, modern fungicides are broadly classified according to their primary target biological processes, providing a basis for understanding fungicide modes of action and their biological targets. In addition, ongoing efforts to develop more precise and selective fungicides are emerging. Taken together, these advances define a path toward the development of safe, specific, and sustainable next-generation fungicides. Here, we review the modes of action of selected commercially available fungicides (**Table 1**) and discuss emerging technologies that enable the development of precise and environmentally sustainable antifungal strategies.

**Table 1:** Representative modes of action of commercially available fungicides.

Mode of action	Chemical group	Specific target	Representative compound	IUPAC name	FRAC groupcode
1. Regulation of nucleic acid synthesis	Phenylamides	RNA polymerase I	metalaxyl	methyl 2-[*N*-(2,6-dimethylphenyl)(methoxy)acetamido]propanoate	4
			benalaxyl-M	methyl N-(phenylacetyl)-N-2,6-xylyl-D-alaninate], and oxadixyl [2-methoxy-N-(2-oxo-3-oxazolidinyl)acet-2’,6’-xylidide	4
	Cyanoacetamide oximes	Nucleic acid related metabolic processes	cymoxanil	(1*E*)-2-(ethylcarbamoylamino)-*N*-methoxy-2-oxoethanimidoyl cyanide	27
	Hydroxypyrimidines	Act as pyrimidine analogues	dimethirimol	5-butyl-2-(dimethylamino)-4-methyl-1*H*-pyrimidin-6-one	8
			ethirimol	5-butyl-2-(ethylamino)-4-methyl-1*H*-pyrimidin-6-one	8
2. Effects on amino acid biosynthetic pathways	Anilinopyrimidines	Inhibit either cystathionine γ-synthase or cystathionine β-lyase in the methionine biosynthetic pathway	pyrimethanil	4,6,-dimethy-N-phenyl-2-pyrimidinamine	9
			cyprodinil	4-cyclopropyl-6-methyl-N-phenylpyrimidin-2-amine	9
		Inhibition of the methionine biosynthetic pathway	mepanipyrim	4-methyl-N-phenyl-6-(1-propynyl)-2-pyrimidinamine	9
3. Modification of signaling cascades	Phenylpyrroles	Interfere with a protein kinase III	fludioxonil	4-(2,2-difluoro-1,3-benzodioxol-4-yl)-1H-pyrrole-3-carbonitrile	12
	Dicarboximides	Osmosensor	iprodione	3-(3,5-dichlorophenyl)-N-isopropyl-2,4-dioxoimidazolidine-1-carboxamide	2
		cAMP-dependent protein kinase	vinclozolin	(RS)-3-(3,5-dichlorophenyl)-5-methyl-5-vinyl-1,3-oxazolidine-2,4-dione	2
4. Impact on mitosis and cell devision	Benzimidazoles	β-tubulin	thiabendazole	2-(4’-thiazolyl)benzimidazole	1
			carbendazim	methyl N-(1H-benzimidazol-2-yl)carbamate	1
5. Control respiration	Pyrimidinamines	Mitochondrial complex I (NADH:ubiquinone oxidoreductase)	diflumetorim	(RS)-5-chloro-N--6-methylpyrimidin-4-ylamine	39
	Pyridine carboxamides	Complex II	boscalid	2-chloro-N-(4’-chlorobiphenyl-2-yl)nicotinamide	7
	Oxathiin carboxamides		carboxin	5’6’-dihydro-2-methyl-1,4-oxathiin-3-carboxanilide	7
	Phenyl benzamides		flutolanil	α,α,α-trifluoro-3’-isopropoxy-o-toluanilide	7
	2,6-dinitroanilines	Uncoupling of the proton gradient across the inner mitochondrial membrane	fluazinam	3-chloro-N-(3-chloro-5-trifluoromethyl-2-pyridyl)-α,α,α-trifluoro-2,6-dinitro-p-toluidine	29
6. Targeting lipids and sterols in membrane compartments	Phenylbenzenes	Linoleic acids in the mitochondrial membrane	dicloran	2,6-dichloro-4-nitroaniline	14
	Thiadiazoles	Phospholipids in the intercellular membranes	etridiazole	5-ethoxy-3(trichloromethyl)-1,2,4-thiadiazole	14
	Triazoles	Inhibits ergosterol biosynthesis	triadimefon	1-(4-chlorophenoxy)-3,3-dimethyl-(1,2,4-triazol-1-yl)-2-butanone	3
	Hydroxyanilides	3-keto reductase	fenhexamid	N-(2,3-dichloro-4-hydroxyphenyl)-1-methylcyclohexanecarboxamide	17

## 2 Modes of action of major fungicidal agents

### 2.1 Regulation of nucleic acid synthesis

Targeting nucleic acid synthesis is important in fungicide development because DNA and RNA production are essential for fungal growth, replication, and gene expression. Inhibiting these processes directly suppresses cell division and limits the rapid growth of pathogenic fungi during infection. Because nucleic acid synthesis is a fundamental and often non-redundant pathway, disruption can impose a high fitness cost on resistant mutants, making it a valuable strategy for resistance management (Anderson, 2005; Lucas et al., 2015). Moreover, although nucleic acid synthesis pathways are conserved across eukaryotes, structural and biochemical differences in fungal enzymes can be utilized for selective targeting, thereby reducing host toxicity and maintaining efficacy (Barba-Aliaga et al., 2021).

Targeting nucleic acid synthesis in fungi can occur at multiple levels, including direct inhibition of transcriptional machinery, as well as indirect regulation of nucleotide metabolism and nucleic acid integrity through chemical analogues. In particular, phenylamide (PA) fungicides constitute a major class that targets ribosomal RNA (rRNA) biosynthesis in oomycetes by inhibiting RNA polymerase I activity, disrupting key developmental processes such as hyphal growth, sporangia formation, and other life cycle stages. Representative compounds, including metalaxyl and benalaxyl-M (FRAC Code 4), share a common mode of action characterized by selective inhibition of RNA polymerase I, leading to suppression of ribosomal RNA synthesis (Kerkenaar, 1981; Sierotzki et al., 2019). This results in arrested mycelial growth, reduced sporulation, and inhibition of zoospore release in important oomycete pathogens such as *Phytophthora infestans* and *Plasmopara* spp. (Zhang Z. et al., 2025).

In addition to transcriptional inhibition, nucleic acid synthesis can also be affected through metabolic interference and analogue-based mechanisms. Cymoxanil (FRAC Code 27) is widely used in agriculture and is associated with interference in nucleic acid-related metabolic processes, leading to impaired fungal development (Kazmirchuk et al., 2024). Hydroxypyrimidine fungicides such as dimethirimol and ethirimol (FRAC Code 8) act as pyrimidine analogues that disrupt nucleic acid metabolism and inhibit fungal growth (Hollomon and Chamberlain, 1981). Taken together, nucleic acid synthesis represents a valuable fungicide target with multiple points of chemical intervention and enables the development of selective compounds with strong potential for disease control and resistance management.

### 2.2 Effects on amino acid biosynthetic pathways

Amino acids are fundamental building blocks for protein synthesis and play essential roles in the pathogenesis of fungi. During host-fungus interactions, these organisms face nutrient limitation, which must be overcome either by activating *de novo* amino acid biosynthetic pathways or by acquiring nutrients from host cells. Inhibition of amino acid biosynthesis, especially branched-chain amino acid synthesis (BCAA) including leucine, isoleucine, and valine, significantly reduces pathogenicity of *Fusarium graminearum* and *Magnaporthe oryzae* (Liu et al., 2014; Que et al., 2020), indicating that amino acid biosynthetic pathways in pathogenic fungi are excellent targets to attenuate virulence.

The anilinopyrimidines are a class of systemic fungicides containing a pyrimidine ring attached to an aniline group within the chemical scaffold. The major derivatives such as pyrimethanil, cyprodinil, and mepanipyrim (FRAC Code 9) are widely used to control mainly Ascomycetes. Pyrimethanil and cyprodinil have been proposed to inhibit either cystathionine γ-synthase which catalyzes the replacement of the succinyl group of activated homoserine with the thiol group of L-cysteine or cystathionine β-lyase which converts L-cystathionine into homocysteine in the methionine biosynthetic pathway in *Botrytis cinerea* and *Penicillium digitatum* (Fritz et al., 2003; Zhang Y. et al., 2021). The anilinopyrimidines are often combined with sterol biosynthetic inhibitors to manage fungal infections in cereals and fruits. These fungicides exhibit strong preventive activity by inhibiting sporulation, early germ tube elongation, and the formation of specialized infection structures such as haustoria. Inhibition of haustoria formation appears to be associated with suppression of the secretion of hydrolytic enzymes including cutinase, pectinase, and cellulase, resulting in their accumulation within fungal cells rather than direct inhibition of the enzymatic activities involved in cell wall degradation required for successful host infection (Miura et al., 1994). They are capable of translocation to the apoplastic regions in leaves where they may also contribute to suppression of later stages of pathogenesis including intercellular mycelial growth (Gisi et al., 2019).

### 2.3 Modification of signaling cascades

Communication between host plants and fungal pathogens is an important component of plant immune responses for discriminating between self and non-self. This dynamic interaction is governed by complex signaling networks that regulate stress perception, cellular adaptation, and defense responses in both organisms. Fungal pathogens perceive host-derived environmental cues and activate conserved stress response pathways, including oxidative stress regulation, nutrient adaptation, and autophagy, which are essential for fungal survival and successful infection. Following contact with plant surface under conditions favorable for infection, fungal spores rapidly germinate and the emerging hyphae penetrate the physical barriers of the cuticle surrounding plant tissues either directly or through the development of appressoria and penetration pegs (Liu et al., 2010; Zhang X. et al., 2021). In response, plants activate innate immune systems for perceiving and responses to pathogen-derived molecules known as pathogen-associated molecular patterns (PAMPs) and effector molecules, initiating defense signaling pathways that restrict pathogen colonization. In compatible interactions, fungal pathogens circumvent the host defense mechanisms by producing and deploying virulence factors that suppress plant immunity (Jones et al., 2024). This process is influenced by physical and chemical cues and is often utilized by fungal pathogens to reprogram transcriptional and metabolic networks required for invasion and colonization (Kou and Naqvi, 2016; Kilaru et al., 2026). Therefore, the inhibition of fungal signaling cascades can compromise the optimal fitness during host infection, resulting in increased susceptibility.

Fungal pathogenicity is tightly linked to conserved stress signaling networks targeted by fungicides. The phenylpyrrole fungicidal agent, fludioxonil (FRAC Code 12), is nonsystemic and has been proposed to interfere with a protein kinase III (PKIII) involved in glycerol synthesis in *Neurospora crassa*. It has been suggested that treatment with fludioxonil leads to the accumulation of glycerol in the mycelium, associated with hypophosphorylation of an unknown downstream protein and subsequent activation of the MAP kinase cascade, promoting glycerol synthesis (Pillonel and Meyer, 1997). Genetic analyses of phenylpyrrole-resistant *N. crassa* strains implicate the histidine kinase Os1p encoded by *NIK1*/*OS-1*, as a key osmosensor required for fungicide sensitivity (Ochiai et al., 2001). It is plausible that the cytoplasmic osmosensor perceives fludioxonil and triggers hyperactivation of the osmotic stress response pathway, particularly the high-osmolarity glycerol (HOG) MAP kinase cascade. This results in excessive glycerol accumulation, leading to cellular swelling and ultimately cell death (Kilani and Fillinger, 2016). Although dicarboximide fungicides are a distinct class from phenylpyrrole fungicidal agents, they affect regulatory components of the same osmotic stress response pathway. Fungicidal effects of a dicarboximide fungicide, iprodione (FRAC Code 2), have been examined in a pathogenic yeast, *Candida albicans*. Similar to fludioxonil, iprodione also stimulates glycerol synthesis in *C. albicans* but not in *Saccharomyces cerevisiae*. According to phenotypic analysis of iprodione-resistant *N. crassa* strains, mutations in histidine kinase *NIK1/OS-1* are responsible for the resistance phenotype, suggesting that insensitivity of the histidine kinase-mediated osmotic stress response to iprodione reduces susceptibility. Consistently, CaNIK1/COS1 in *C. albicans* is a homolog of NIK1/OS-1 of *N. crassa*, supporting a conserved role for this osmosensing apparatus in regulating glycerol synthesis in response to fungitoxic stress (Ochiai et al., 2002). Additionally, previous studies have demonstrated that cAMP-dependent protein kinase (PKA) is implicated in resistance to vinclozolin (FRAC Code 2) in *Ustilago maydis*, although its precise mode of action remains unclear (Orth et al., 1995). While vinclozolin does not directly affect PKA kinase activity, mutations in *UBC1* encoding the regulatory subunit of PKA are associated with the vinclozolin resistance. Moreover, vinclozolin-resistant *ubc1* mutants exhibit osmotic sensitivity, indicating that cAMP signaling pathway linked to the osmotic stress response appears to be a responsive component targeted by vinclozolin (Ramesh et al., 2001). Together with the involvement of NIK1/OS-1-mediated signaling targeted by fludioxonil and iprodione, these findings indicate that the cAMP-dependent signaling cascade is interconnected with the HOG MAP kinase stress response network. Crosstalk between growth-regulatory cAMP signal cascade and stress-adaptive HOG signaling coordinates fungal fitness. Fungicides disrupt this balance by perturbing the regulatory interplay between these signaling pathways.

### 2.4 Impact on mitosis and cell division

Spore germination and expansion, hyphal growth, and formation of infection structures rely on fungal mitosis and cell division (Saunders et al., 2010; Steinberg et al., 2017). Disruption of these processes causes growth arrest, leading to failure of infection and cell death. Accordingly, mitosis and cell division represent core antifungal vulnerabilities, as fungicides targeting these processes are highly effective against actively growing fungi and exhibit broad-spectrum activity during early stages of infection. Most mitosis-targeting fungicides do not directly inhibit DNA replication but instead disrupt the mitotic spindle, preventing chromosome segregation (Davidse and Flach, 1978; Davidse, 1986).

Thiabendazole and carbendazim (FRAC Code 1) are systemic fungicides classified within the methyl benzimidazole carbamate (MBC) class. They directly bind to β-tubulin and prevent microtubule polymerization. The loss of microtubule integrity disrupts assembly of the mitotic spindle, resulting in mitotic arrest in the hyphae of *Aspergillus nidulans* (Davidse and Flach, 1978) due to impaired kinetochore-microtubule attachment and chromosome segregation at the metaphase plate. In addition, disruption of spindle formation activates cell cycle checkpoint control systems such as the spindle assembly checkpoint (Musacchio and Salmon, 2007). The fungicide application delays anaphase and triggers mitotic arrest rather than directly inhibiting DNA synthesis or checkpoint regulators.

### 2.5 Control respiration

Mitochondrial respiration is a major target of fungicides, with multiple classes disrupting distinct components of oxidative phosphorylation. Compared with vertebrates, most fungi possess branched respiratory chains in which alternative NADH dehydrogenases confer metabolic plasticity and enable adaptation to environmental stresses. Notably, type II NADH dehydrogenases (NDH-2) catalyze rotenone-insensitive NADH oxidation and bypass respiratory Complex I without proton translocation, resulting in reduced ATP production per NADH compared with canonical mitochondrial respiration (Joseph-Horne et al., 2001; Gonçalves and Videira, 2015). Although this alternative respiratory pathway is less energy efficient, it maintains electron transport activity and mitochondrial redox homeostasis, thereby enhancing fungal adaptation to oxidative and metabolic stress. Natural products such as piericidin A and rotenone inhibit mitochondrial Complex I, disrupting electron transport and energy production. Although these compounds exhibit antifungal potential, they are not currently assigned FRAC codes because they have not been developed as registered agricultural fungicides. Furthermore, the metabolic flexibility of fungal respiratory systems, including the ability to utilize alternative NADH oxidation pathways, can reduce sensitivity to Complex I inhibitors and enable fungal survival under respiratory stress (Singer and Ramsay, 1994; Lümmen, 1998).

A small molecule has been reported to inhibit NDH-2. HDQ is a high-affinity competitive inhibitor of the ubiquinone-binding site of NDH-2 in *Yarrowia lipolytica*, indicating a promising candidate for managing fungal infection (Eschemann et al., 2005). It is worth noting that HDQ-1 has not been registered as a fungicide. For respiratory chain-targeting fungicides, diflumetorim (FRAC Code 39) is a pyrimidinamine fungicide to control powdery mildew and rust diseases in ornamental plants. It inhibits mitochondrial complex I (NADH:ubiquinone oxidoreductase) which catalyzes NADH oxidation coupled to proton translocation across the inner mitochondrial membrane, leading to impaired fungal respiration and cell death (Li et al., 2023). In the case of complex II-targeting fungicides, boscalid, carboxin, and flutolanil (FRAC Code 7) inhibit the activity of succinate dehydrogenase (SDH), a key enzyme linking the tricarboxylic acid (TCA) cycle and the mitochondrial electron transport chain. SDH is a multi-subunit complex composed of a flavoprotein, an iron-sulfur protein, and membrane-anchoring cytochrome b subunits, of which the former two subunits form the catalytic core responsible for the oxidation of succinate to fumarate. SDH inhibitors bind to the ubiquinone binding pocket located within the membrane-anchoring subunits of the SDH complex, blocking electron transfer to ubiquinone and inhibiting fungal respiration (Li et al., 2021). In contrast to complex I and II inhibitors, fluazinam (FRAC Code 29) targets a distinct respiration mechanism. It is an uncoupler of oxidative phosphorylation, dissipating the proton gradient required to generate the protonmotive force across the inner mitochondrial membrane, rather than directly inhibiting electron transport chain complexes. The collapse of the proton gradient inhibits ATP synthesis, resulting in mitochondrial dysfunction and energy depletion.

### 2.6 Targeting lipids and sterols in membrane compartments

The cellular membrane is a phospholipid bilayer with selective permeability that separates the intracellular and intraorganellar environments from their surroundings. It serves as a dynamic regulatory hub that preserves cellular structural integrity and primarily governs water potential, material transport, and signal transduction mediated by membrane-associated transporters and receptors embedded within a complex matrix of lipids, sterols, and carbohydrate macromolecules. Given that disruption of individual membrane components has deleterious effects on cellular homeostasis, signaling cascades and survival, many fungicidal agents target these components to impair the growth and pathogenicity of fungal pathogens.

Fungicidal scaffolds containing aromatic hydrocarbon moieties can disrupt fungal membrane systems. Dicloran (FRAC Code 14) has been examined as a fungicide against several soil fungi such as *Botryotrichum piluliferum, Clonostachys rosea f. rosea, Humicola fuscoatra, Sepedonium chrysospermum*, and *Trichoderma viride* (Kuthubutheen and Pugh, 1979). To evaluate its cellular effects, dicloran treatment of rat liver cells has been shown to induce morphological changes including mitochondrial swelling (Bachmann et al., 1971); however, single-dose exposure did not affect mitochondrial function (Gallo et al., 1976). In addition, its phototoxicity is linked to interactions with linoleic acid, suggesting a role for radical-mediated membrane damage (Boscá et al., 1998). These findings indicate that dicloran disrupts mitochondrial membrane integrity, either through physicochemical interactions with lipid membrane components or by inducing lipid peroxidation, both of which compromise membrane potential, permeability, and membrane-associated signaling pathways. Consistent with this mechanism, another fungicide with aromatic hydrocarbon moieties, etridiazole (FRAC Code 14), has been shown to inhibit the growth of a soil saprotrophic fungus, *Mucor mucedo*. Etridiazole stimulates the hydrolysis of membrane-bound phospholipids, resulting in the accumulation of free fatty acids and lysophosphatides. This lipid hydrolysis and oxidation primarily occur in the inner mitochondrial membrane rather than in other intracellular membranes. Such lipid metabolism-mediated toxicity accounts for the fungicidal mechanism of etridiazole (Radzuhn and Lyr, 1984).

Sterols also play pivotal roles in maintaining membrane fluidity and permeability. Vertebrate and plant cells contain distinct sterols with cholesterol predominating in vertebrates and campesterol, sitosterol, and stigmasterol in plants, whereas ergosterol is the predominant sterol component of fungal membranes and is essential for fungal growth and viability (Kazan and Gardiner, 2017). Triadimefon (FRAC Code 3) is a systemic triazole fungicide applied to foliage for controlling Uromyces, Ascomycetes, and Basidiomycetes (Buchenauer, 1979; Pring, 1984). Triadimefon strongly inhibits ergosterol biosynthesis by blocking C-14 demethylation of the sterol carbon skeleton, and the resulting accumulation of sterol precursors causes dysfunction of fungal membranes, indicating that ergosterol synthesis is the primary target of the fungicide in sporidia of *Ustilago avenae* (Buchenauer, 1977). Similarly, the hydroxyanilide derivative fenhexamid (FRAC Code 17) targets 3-keto reductase, an enzyme involved in C-4 demethylation during ergosterol biosynthesis in the grey mold *B. cinerea*. Fenhexamid application inhibits germ-tube elongation and mycelia growth, but not the germination of high-sterol-containing spores of *B. cinerea* due to the accumulation of ergosterol biosynthetic intermediates, including 3-keto compounds, 4-α-methylfecosterone, fecosterone, and episterone. Inhibition of 3-keto reductase reduces ergosterol levels and leads to the accumulation of these intermediates in membrane compartments, resulting in membrane leakage, loss of membrane integrity, and toxic effects on fungal pathogens (Debieu et al., 2001).

## 3 Discussion

Current fungicides are increasingly challenged by the rapid emergence of resistant pathogen populations, environmental persistence, and growing regulatory pressure to reduce chemical inputs (Williams et al., 2024). The repeated application of fungicides with similar modes of action has accelerated the selection and spread of resistant strains among many economically important fungal pathogens, significantly reducing the long-term effectiveness of existing disease management strategies (Hahn, 2014; Burandt et al., 2024; Islam et al., 2024). In addition, concerns regarding environmental accumulation, non-target effects, and regulatory restrictions have created an urgent need for safer and more sustainable crop protection solutions. Consequently, fungicide discovery has shifted from traditional empirical screening toward target-based, mechanism-driven approaches that prioritize the identification of novel molecular targets, improved pathogen selectivity, reduced environmental impact, and compatibility with integrated disease management programs.

Recent technological advances are fundamentally transforming fungicide discovery by making the process more predictive, efficient, and data-driven. AI-driven fungicide design increasingly leverages machine learning to integrate diverse large-scale chemical, genomic, biological, environmental, and agronomic datasets, enabling prioritization of candidate molecules, prediction of antifungal activity and toxicity, and optimization of lead compounds before extensive experimental validation (Shastri et al., 2025; Hu et al., 2026). Computational approaches, including molecular docking, molecular simulations, and quantitative structure-activity relationship (QSAR) modeling (Paggi et al., 2024; Gálvez-Llompart et al., 2026), combined with advances in protein structure prediction, structural biology, and high-throughput biochemical screening, enable the identification of compounds that interact with pathogen-specific targets while minimizing potential off-target effects. These integrated approaches accelerate the discovery of novel fungicide candidates by improving target validation, reducing screening costs, and shortening the overall development pipeline.

In parallel, researchers are expanding the chemical and biological resources for sustainable crop protection by exploring natural products (Ben El Caid et al., 2026), secondary metabolites (Martínez et al., 2017), and bioactive compounds derived from extremophilic (Yarzábal Rodríguez et al., 2024; Miloudi-Agha and Kebdani, 2026) and marine microorganisms (Menaa et al., 2020). These resources provide unique chemical scaffolds and previously unexplored mechanisms of action that may overcome limitations associated with existing fungicide classes. Advances in genome mining, metabolomics, biosynthetic gene cluster analysis, and synthetic biology have further enhanced the ability to identify, characterize, and engineer novel bioactive compounds for agricultural applications. At the same time, biological fungicides derived from beneficial microorganisms, RNA interference (RNAi)-based crop protection technologies, and elicitor-induced plant immunity are emerging as sustainable alternatives that provide highly specific disease control while reducing environmental persistence and unintended effects on non-target organisms (Cantrell et al., 2012; Fletcher et al., 2020; Chen et al., 2024).

The identification of novel biological targets has become a central strategy for overcoming the limitations of current fungicides. The modes of action of previously described fungicidal compounds (**Figure 1**) provide insight into how advances in target discovery are being translated into the development of next-generation fungicides. Among the emerging biological processes being explored, fungal autophagy has attracted considerable attention because of its essential roles in cellular homeostasis, nutrient recycling, stress adaptation, and pathogen virulence. Genetic and biochemical studies have demonstrated that disruption of core autophagy components suppresses critical infection-related processes, including spore germination, mycelial growth, appressorium formation, and pathogenicity in several devastating fungal pathogens (Liu et al., 2010, 2019). Building on these findings, a recently developed bioluminescence resonance energy transfer (BRET)-based high-throughput screening platform enabled the identification of ebselen and its analogs as autophagy inhibitors in *B. cinerea* and *M. oryzae*, demonstrating the feasibility of target-based biochemical screening for discovering fungicides with novel modes of action (Woo et al., 2024).

**Figure 1:**
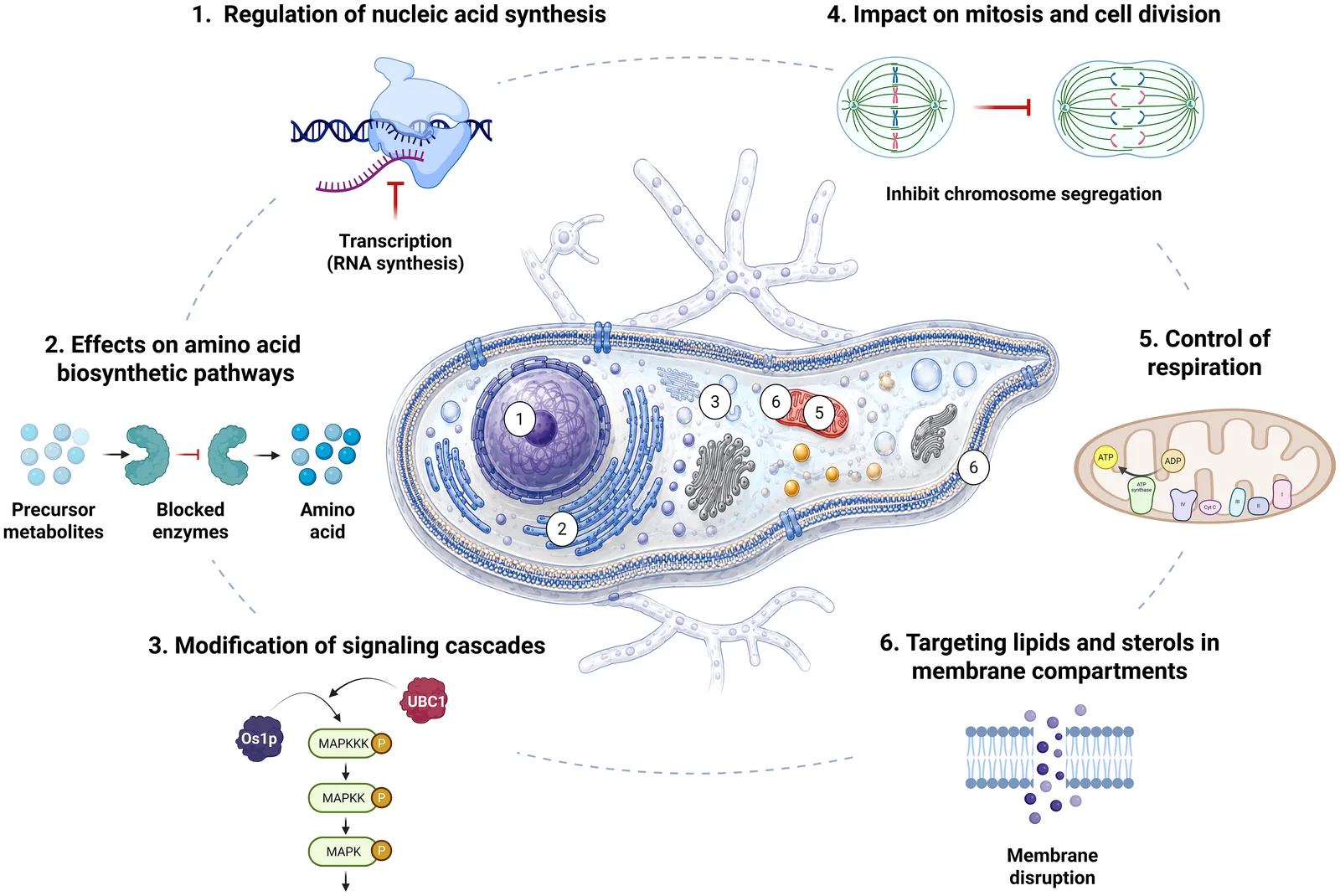
Overview of the principal modes of action of fungicides against phytopathogenic fungi. The major cellular processes targeted by fungicides are described in this review. Target pathways include nucleic acid synthesis, amino acid biosynthesis, signal transduction, mitosis, respiration, and membrane lipid/sterol homeostasis. Encircled numbers indicate target organelles.

Beyond the discovery of new antifungal molecules, advances in nanotechnology and precision agriculture are improving the practical performance and sustainability of fungicides under field conditions (Farías-Vázquez et al., 2024). Nano-biopesticides, including biopolymer-based nanoparticles (e.g., chitosan and zein) and metallic nanoparticles (e.g., silver, gold, titanium dioxide, and zinc oxide), enhance fungicide efficacy through improved stability, targeted delivery, increased bioavailability, and controlled release while reducing pesticide application rates, off-target losses, environmental contamination, and impacts on non-target organisms (Jitendra et al., 2025). For example, Ag@TiO_2_ and Ag@SeO_2_ nanoparticles synthesized using *Beta vulgaris* extract exhibited strong antifungal activity against *Rhizoctonia solani* and *Sclerotinia sclerotiorum*, demonstrating the potential of plant extract-mediated synthesis for developing environmentally sustainable antifungal nanomaterials (Elattar et al., 2024). In addition, nanozyme-based materials have emerged as a new class of nanoparticle-enabled antifungal agents that further improve foliar adhesion, compound persistence, and catalytic antimicrobial activity under field conditions (Wang et al., 2022). Twinned Cu-Fe nanozyme platforms have demonstrated durable suppression of *B. cinerea* through sustained peroxidase-like catalytic activity and endogenous reactive oxygen species generation while maintaining long-term stability under oxidative conditions (Liu et al., 2025). Complementing these advances, precision agriculture technologies — including AI-based disease forecasting, unmanned aerial vehicles equipped with hyperspectral and multispectral imaging (Zhang S. et al., 2025), satellite remote sensing, Internet of Things (IoT)-based monitoring systems — enable earlier disease detection and more precise fungicide application, improving disease control efficiency while reducing unnecessary chemical inputs and environmental impacts (Delfani et al., 2024; Xing et al., 2026).

In conclusion, next-generation fungicide development is shifting from conventional broad-spectrum chemical control toward precision, target-based strategies that prioritize novel biological targets, enhanced selectivity, and environmental sustainability. The integration of AI-assisted discovery, computational modeling, high-throughput screening, natural-product exploration, biological control, RNAi technologies, advanced nanomaterial-based formulations, and precision agriculture is accelerating the emergence of more effective, durable, and environmentally responsible crop protection solutions. Taken together, these advances establish a comprehensive framework for designing sustainable fungicides capable of overcoming the evolving challenges posed by plant pathogenic fungi.
